# Case report: Alpelisib-induced Stevens–Johnson syndrome

**DOI:** 10.3389/fonc.2022.954027

**Published:** 2022-09-28

**Authors:** Christine Jane Kurian, Akshay Desai, William Rafferty, Ahmed Kamel Abou Hussein

**Affiliations:** ^1^ Department of Hematology and Medical Oncology, MD Anderson Cancer Center at Cooper University Healthcare, Camden, NJ, United States; ^2^ Department of Internal Medicine, Cooper University Healthcare, Camden, NJ, United States; ^3^ Department of Pathology, Cooper University Healthcare, Camden, NJ, United States

**Keywords:** breast cancer, metastatic breast cancer, alpelisib, Stevens Johnson syndrome (SJS), Stevens Johnson Syndrome

## Abstract

**Background:**

Alpelisib is a recently approved treatment for hormone receptor-positive, HER2-negative, PIK3CA-mutated advanced breast cancer. It has been associated with alopecia and rash, but there are no documented cases of Stevens–Johnson Syndrome (SJS) associated with this drug. Here, we detail the first case of SJS associated with alpelisib.

**Case description:**

Our patient is a 60-year-old woman with a past medical history of metastatic hormone receptor-positive (ER+ 80% and PR+ 1%), HER2-negative metastatic breast cancer who presented with acute odynophagia, fevers, and diffuse body rash after receiving her first doses of alpelisib and fulvestrant in the preceding days. She presented to the emergency department after developing a whole-body rash and severe ulceration of her buccal mucosa. She was started on methylprednisolone with remarkable improvement in symptoms.

**Conclusion:**

This case report details the only report of SJS following alpelisib treatment. Immediate cessation of drugs and initiation of steroids are the cornerstone of treatment. Patients who experience such side effects will have to be monitored closely for long-term sequelae associated with SJS, including cutaneous, ocular, and oral sequelae, all of which can profoundly affect the quality of life for cancer patients.

## Introduction

As therapies continue to evolve for the treatment of metastatic breast cancer, clinicians must be aware of new possible adverse effects that they may encounter. Alpelisib is a novel oral treatment for advanced hormone receptor-positive, HER2-negative, PIK3CA-mutated breast cancer. All-grade rash related to alpelisib was reported in 53.9% of patients and grade 3 rash in 20.1% in the landmark SOLAR1 trial, which resulted since the approval of this medication (1). This is an on-target side effect. Here, we discuss the first reported case of Stevens–Johnson syndrome likely precipitated by alpelisib.

## Case description

A 60-year-old woman with a past medical history of metastatic hormone receptor-positive (ER+ 80% and PR+ 1%), HER2-negative breast cancer with pulmonary, hepatic, and osseous metastases presented to the emergency department with acute odynophagia, fevers, and diffuse body rash. She had started fulvestrant (500 mg intramuscular (IM) injection) 15 days prior and alpelisib (300 mg daily) 19 days prior to presentation. The patient was not previously taking any antibiotics or CYP3A4 inhibitors prior to presentation. Her symptoms started 4 days prior to presentation when she noticed oral lesions initially with mild erythema and a small ulcer on the inner lower lip mucosa. The ulcers increased in size and number, and she was febrile to 100.7°F, which prompted a call to her primary care physician. She was prescribed magic mouthwash, which worsened her symptoms. She then noticed an erythematous, non-pruritic rash starting on her chest and spreading to her back and presented to the ED. She was noted to have worsening oral pain, sloughing, severe odynophagia, and dysphagia.

## Diagnostic assessment

**Figure 1:**
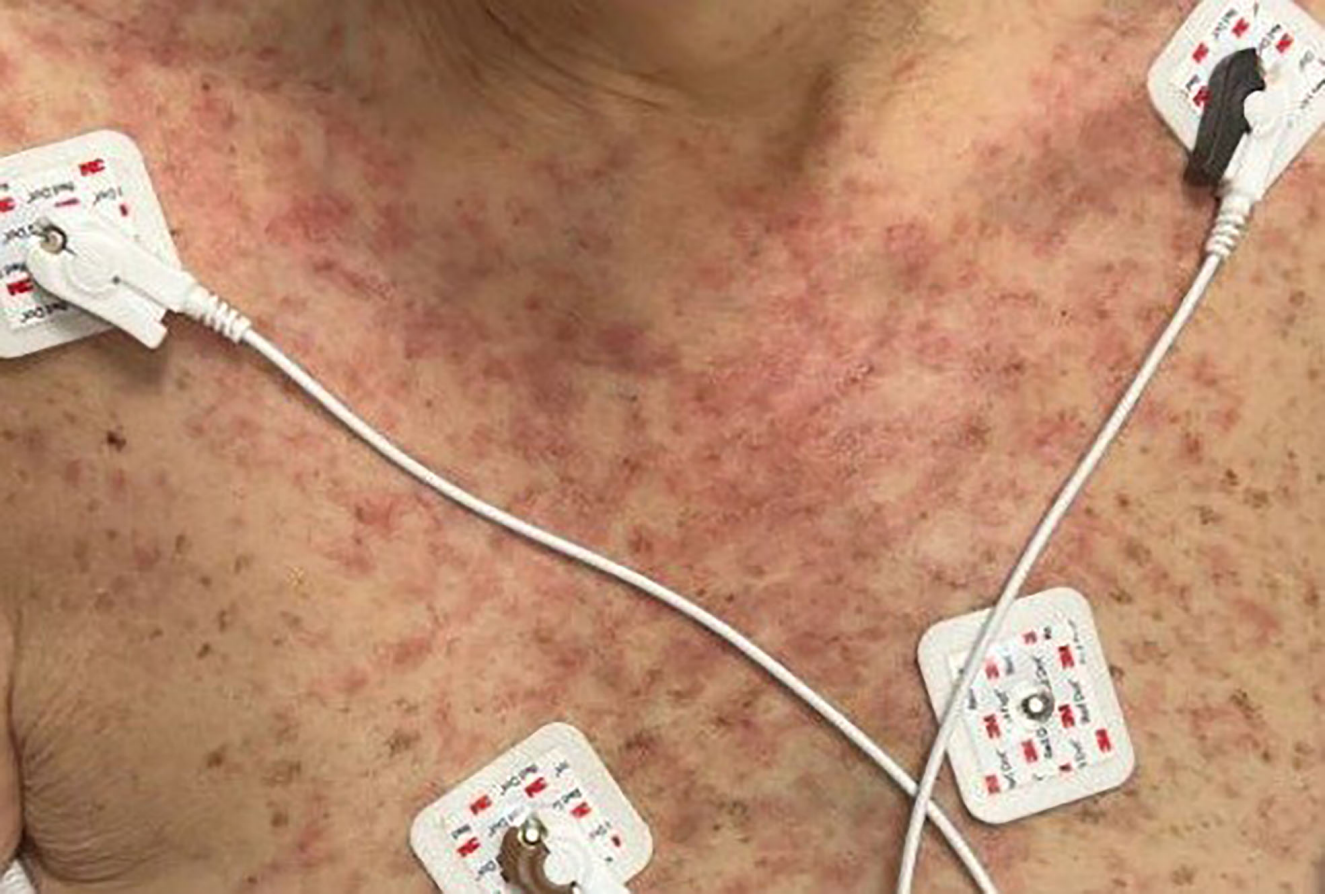
Morbiliform rash and blanching erythema on the back.

**Figure 2:**
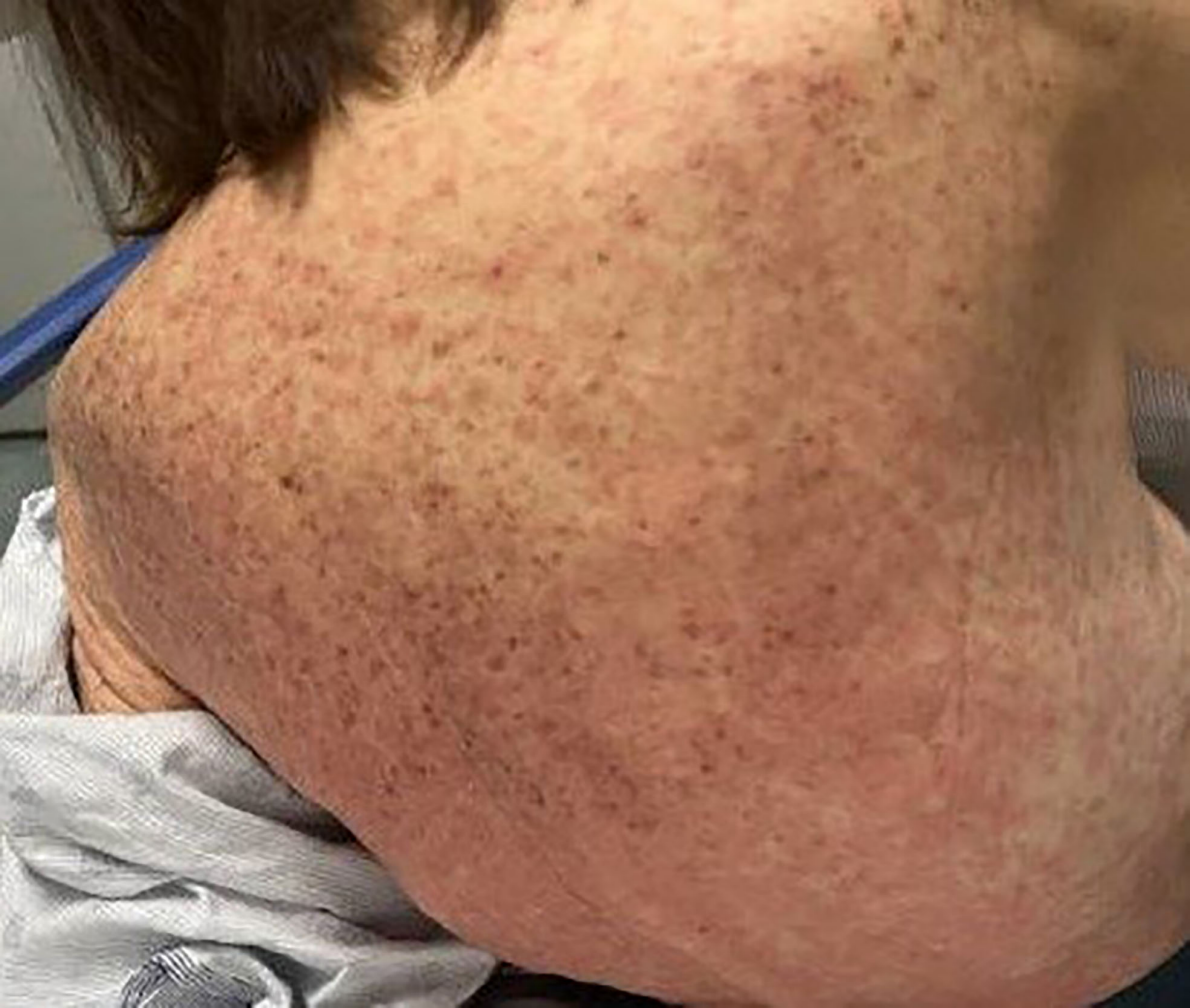
Morbilliform rash and blanching erythema on the trunk.

**Figure 3:**
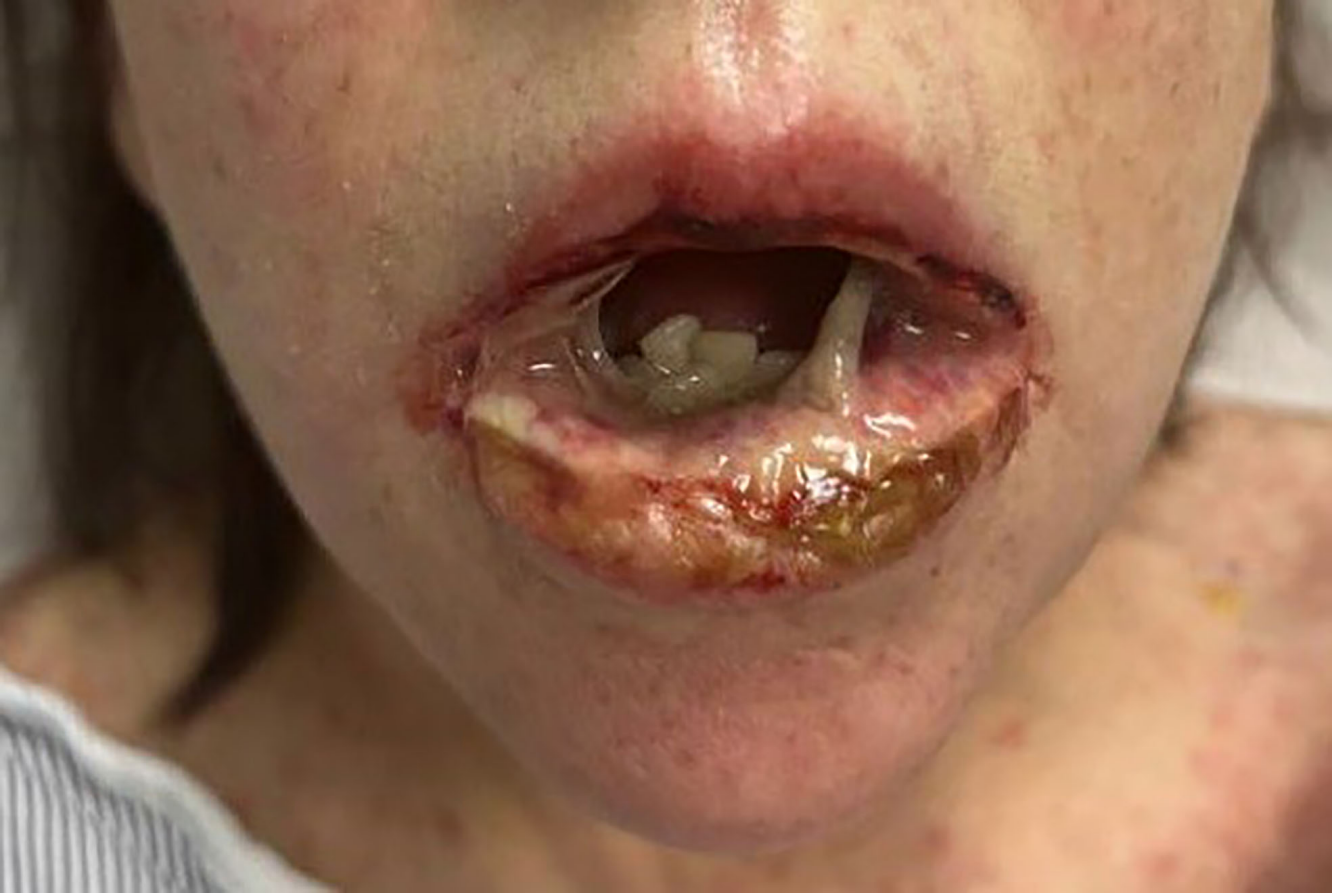
morbiliform rash Exudative and hemorrhagic sloughing of the lips and buccal mucosa.

On admission, she was febrile to 100.7°F and tachycardic with a heart rate of 108. She was normotensive. She noted no nausea, vomiting, chest pain, shortness of breath, and abdominal pain. Complete blood count showed leukocytosis at 13,100. Physical exam was notable for exudative and hemorrhagic sloughing of the lips and buccal mucosa and morbilliform and blanching erythema on the trunk with a superficial erosion on the chest (**Figures 1**–**3**). She was also noted to have tearing in both eyes. She was evaluated by ophthalmology, and the evaluation showed no change in vision with the anterior segment unremarkable and conjunctiva with trace hyperemia and injection without significant discharge or conjunctivitis. No symblepharon formation, episcleritis, or iritis was observed upon examination. On genital exam, she was noted to have erythema localized to the vagina.

Differential diagnoses included Stevens–Johnson Syndrome (SJS)/toxic epidermal necrolysis (TEN), paraneoplastic pemphigus, and reactive infectious mucocutaneous eruption (RIME). However, given the extensive skin sloughing of her lips and oral mucosa, the leading diagnosis was SJS/TEN.

**Figure 4:**
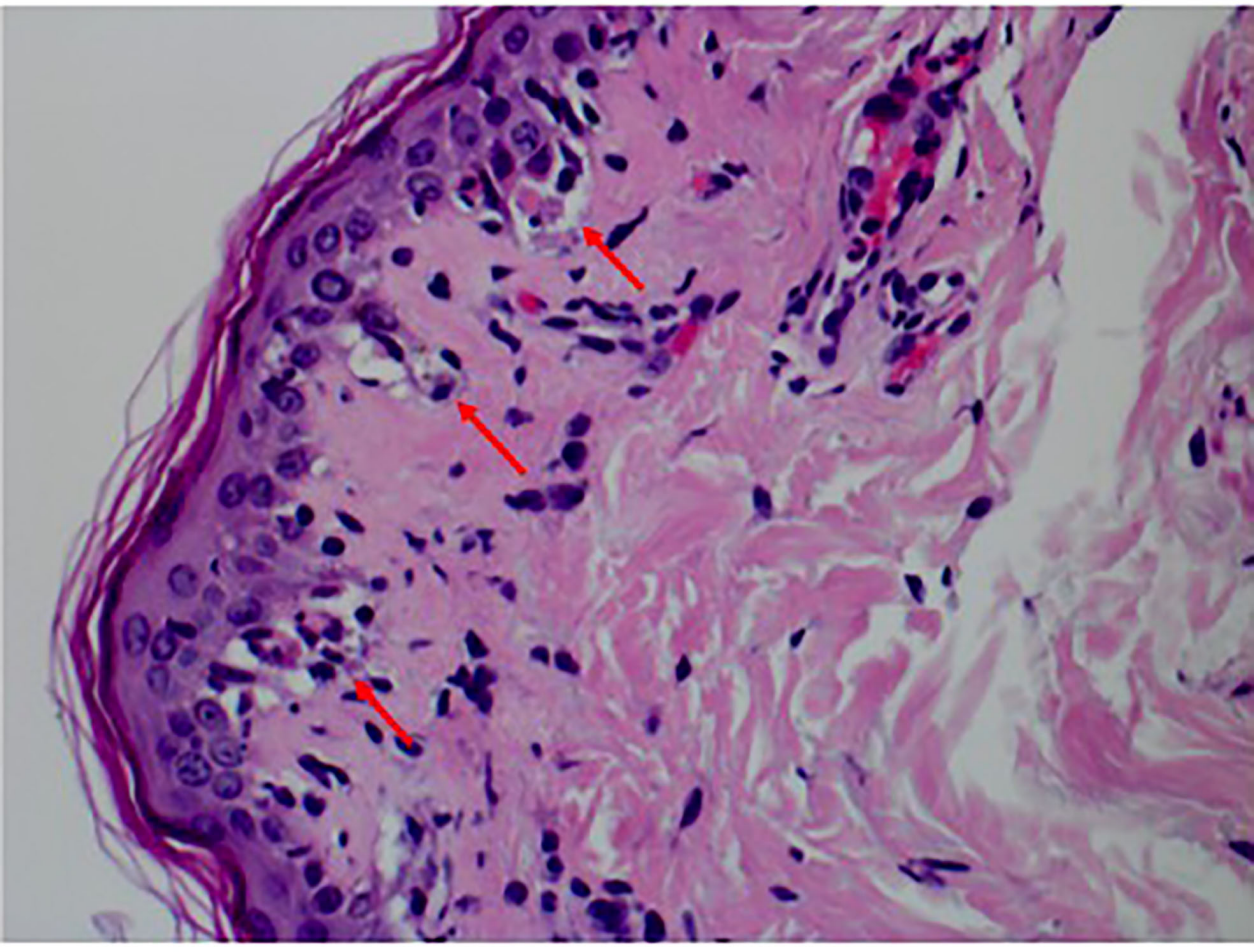
Punch biopsy pathology showing interface dermatitis with basal layer vacuolization, several apoptotic keratinocytes, and a sparse lymphocytic infiltrate.

**Figure 5:**
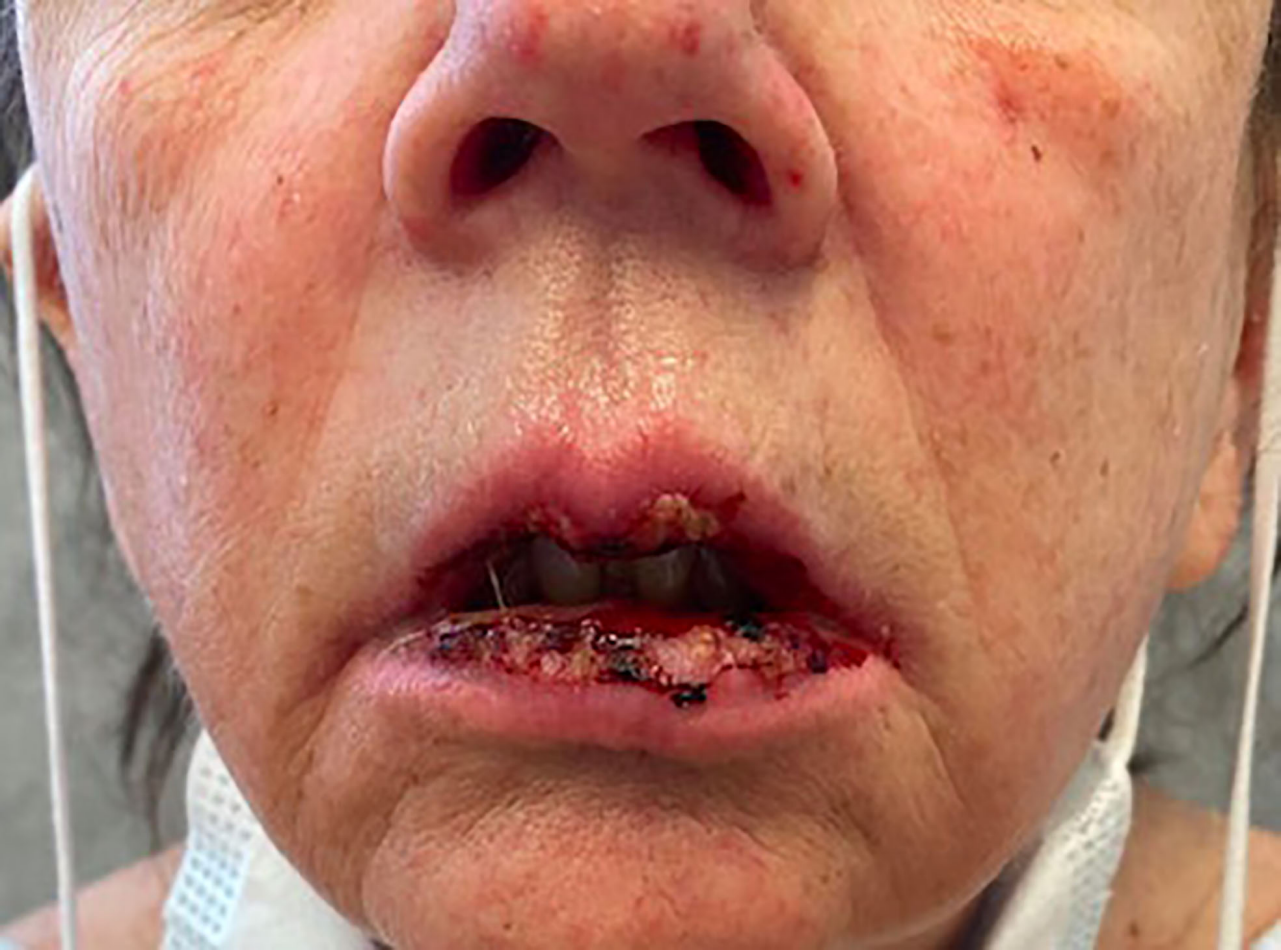
Patient’s mouth 11 days after initial presentation.

**Figure 6:**
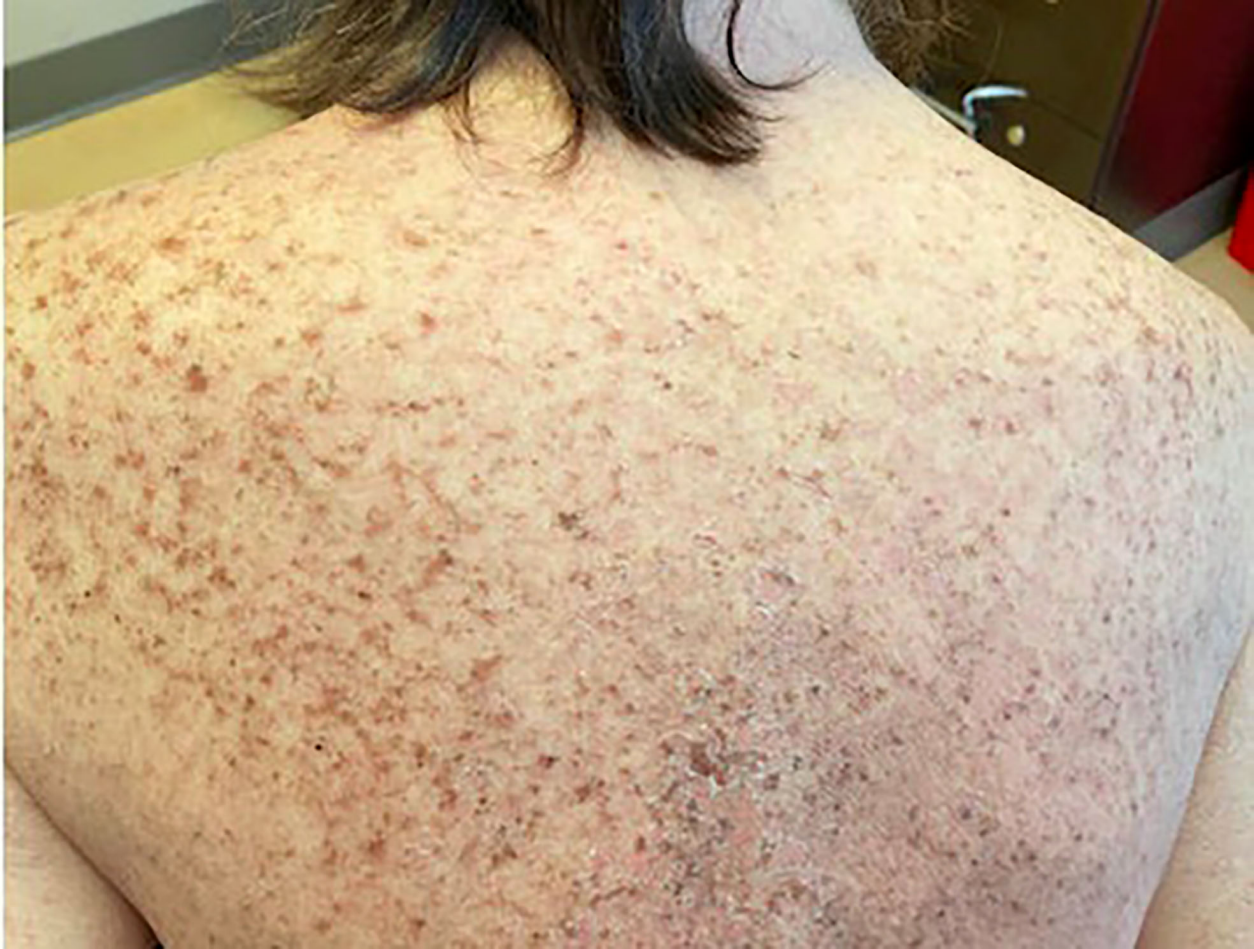
Patient’s back 11 days after initial presentation.

A punch biopsy of the back showed features consistent with mild interface dermatitis with basal layer vacuolization and scattered necrotic keratinocytes consistent with the erythema multiforme spectrum of disorders (**Figure 4**). Both alpelisib and fulvestrant were stopped. She was started on 1 mg/kg of methylprednisolone daily, and her symptoms improved markedly (**Figures 5**, **6**). She was then transitioned to oral steroids with a regimen of 60 mg of prednisone daily for 7 days, then 40 mg of prednisone daily for 7 days, and then 20 mg of prednisone daily for 7 days. She had no long-term sequelae of SJS. Systemic treatment was held until her steroid taper was completed. Of note, she later tolerated subsequent fulvestrant therapy without any side effects.

## Discussion

SJS and TEN represent a spectrum of febrile, mucocutaneous drug-induced reactions. Annually, the incidence of SJS in adults in the United States is estimated to be approximately nine cases per million per year (2). Cases of SJS/TEN have often been associated with various types of antibiotics. These include sulfonamides, tetracyclines, and cephalosporins (3–5). Cases involving other medications including allopurinol, lamotrigine, and imidazole antifungals have also been seen (6–8). The distinction between SJS and TEN is based on the surface area, with SJS being diffuse and involving less than 10% of the total body surface area. It often manifests with widespread erythematous or purpuric macules or flat atypical target-shaped lesions. Conversely, TEN involves greater than 30% of body surface area and may present without any discrete lesions. Skin findings between 10% and 30% are classified as SJS/TEN overlap syndrome.

Alpelisib is indicated for the treatment of advanced or metastatic hormone receptor-positive (HR+), HER2-negative, and PIK3CA-mutated breast cancer, given in combination with fulvestrant (1). It functions *via* the inhibition of phosphatidylinositol-3-kinase (PI3K), primarily acting through the inhibition of PI3K-alpha (9). This facilitates an increase in estrogen receptor transcription, providing new receptor targets for fulvestrant. Although common dermatologic manifestations including alopecia and rash have been associated with alpelisib, serious dermatologic adverse effects such as SJS have been rare (10, 11). While there are additional risk factors that can predispose a patient to SJS/TEN, such as certain human leukocyte antigens (HLAs) or documented cross-reactivity to other medications, our patient did not have such a history that could explain her symptoms. SJS has been seen previously with older chemotherapeutic agents including bleomycin and thalidomide, EGFR inhibitors such as afatinib and cetuximab, and immunotherapeutic agents including nivolumab and pembrolizumab (12). There are no other reported cases of SJS following alpelisib. In addition, this patient’s timeline of symptoms would align with the expected onset of SJS following a new medication, as symptoms often develop within 1–2 weeks of a new treatment.

## Conclusion

This case report details the only report of SJS following alpelisib treatment. The patient tolerated fulvestrant treatment after this incident without reaction. Immediate cessation of the drug is required. Further therapy may involve steroids, cyclosporine, etanercept, or intravenous immunoglobulin (IVIG). Patients who experience such side effects will have to be monitored closely for long-term sequelae associated with SJS, including cutaneous, ocular, and oral sequelae, all of which can profoundly affect the quality of life of cancer patients.

## Data availability statement

The original contributions presented in the study are included in the article/**Supplementary Material**. Further inquiries can be directed to the corresponding author.

## Author contributions

CK and AD wrote the manuscript. AH reviewed and edited the manuscript. WR helped with the pathology slide and explanation. All authors contributed to the article and approved the submitted version.

## Conflict of interest

The authors declare that the research was conducted in the absence of any commercial or financial relationships that could be construed as a potential conflict of interest.

## Publisher’s note

All claims expressed in this article are solely those of the authors and do not necessarily represent those of their affiliated organizations, or those of the publisher, the editors and the reviewers. Any product that may be evaluated in this article, or claim that may be made by its manufacturer, is not guaranteed or endorsed by the publisher.
