# Supplementary figures and images for: Case report: Alpelisib-induced Stevens–Johnson syndrome
Source: Front Oncol. 2022 Sep 28;12:954027. doi: 10.3389/fonc.2022.954027 (PMC9554210; doi:10.3389/fonc.2022.954027)

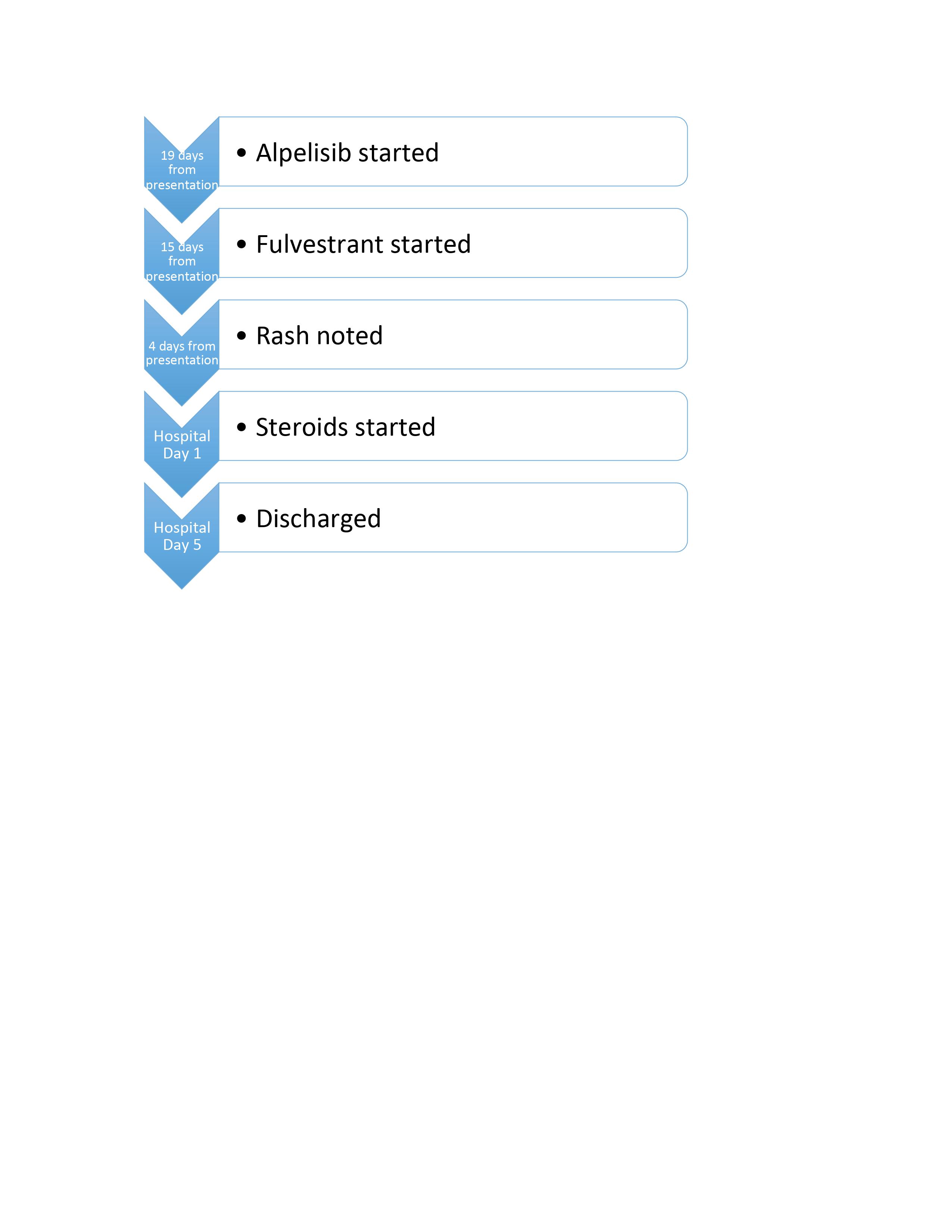

Supplement: Supplementary file 1 [file Image_1.jpeg]
